# Wave-slope soaring of the brown pelican

**DOI:** 10.1186/s40462-021-00247-9

**Published:** 2021-03-22

**Authors:** Ian A. Stokes, Andrew J. Lucas

**Affiliations:** 1 Dept. of Mechanical and Aerospace Engineering, University of California, San Diego, 9500 Gilman Drive, La Jolla, CA 92037, USA; 2 Scripps Institution of Oceanography, University of California, San Diego, 8622 Kennel Way, La Jolla, CA 92037, USA

**Keywords:** Soaring, Wave-slope soaring, Dynamic soaring, Seabird, Pelican, Wave-induced wind, Soliton, Cost-effective flight, Ocean surface gravity waves, Theoretical model

## Abstract

**Background:**

From the laboratory at Scripps Institution of Oceanography, it is common to see the brown pelican (*Pelecanus occidentalis*) traveling along the crests of ocean waves just offshore of the surf-zone. When flying in this manner, the birds can travel long distances without flapping, centimeters above the ocean’s surface. Here we derive a theoretical framework for assessing the energetic savings related to this behavior, ‘wave-slope soaring,’ in which an organism in flight takes advantage of localized updrafts caused by traveling ocean surface gravity waves.

**Methods:**

The energy cost of steady, constant altitude flight in and out of ground effect are analyzed as controls. Potential flow theory is used to quantify the ocean wave-induced wind associated with near-shoaling, weakly nonlinear, shallow water ocean surface gravity waves moving through an atmosphere initially at rest. Using perturbation theory and the Green’s function for Laplace’s equation in 2D with Dirichlet boundary conditions, we obtain integrals for the horizontal and vertical components of the wave-induced wind in a frame of reference moving with the wave. Wave-slope soaring flight is then analyzed using an energetics-based approach for waves under a range of ocean conditions and the body plan of *P. occidentalis.*

**Results:**

For ground effect flight, we calculate a ∼15 - 25% reduction in cost of transport as compared with steady, level flight out of ground effect. When wave-slope soaring is employed at flight heights ∼2m in typical ocean conditions (2m wave height, 15s period), we calculate 60-70% reduction in cost of transport as compared with flight in ground effect. A relatively small increase in swell amplitude or decrease in flight height allows up to 100% of the cost of transport to be offset by wave-slope soaring behavior.

**Conclusions:**

The theoretical development presented here suggests there are energy savings associated with wave-slope soaring. Individual brown pelicans may significantly decrease their cost of transport utilizing this mode of flight under typical ocean conditions. Thus wave-slope soaring may provide fitness benefit to these highly mobile organisms that depend on patchy prey distribution over large home ranges.

## Background

Some birds are able to fly with little flapping by exploiting energy present in the ambient wind-field [1–5]. When these energy gains are great enough to offset the cost of flight, the phenomenon is known as ‘soaring’ [1, 6]. As an energy efficient means of searching for prey or travelling long distances, soaring behaviors are widespread in avians and have demonstrated ecological significance [5, 7–9].

Soaring behaviors in general take advantage of the structure and variability of the fluid flow in the lower atmosphere [2, 4]. For example, when the desert floor and the still air just above is heated by the midday summer sun, vigorous thermal convection can occur. ‘Thermal soaring’ is the familiar behavior associated with catching these updrafts, and is used to gain altitude and locate prey from long distances [2, 4]. The moving atmosphere impinging on raised topography also can create strong vertical flows. ‘Slope soaring’ takes advantage of updrafts that are created by the vertical redirection of airflow over cliffs and steep hills [2, 4].

Soaring behaviors are not limited to localized convection or the presence of topographic obstacles. In the windswept mid- and high-latitude open ocean, seabirds use the vertical shear of wind within the turbulent atmospheric boundary layer to gain energy in a behavior known as ‘dynamic soaring’ [7, 8, 10, 11]. The wandering albatross can circumnavigate the globe, rarely flapping their wings, by employing this technique [7, 8]. The potential for using vertical shear in horizontal winds to power continuous flight was first recognized by Leonardo da Vinci in the 16th century [9].

**Fig. 1:**
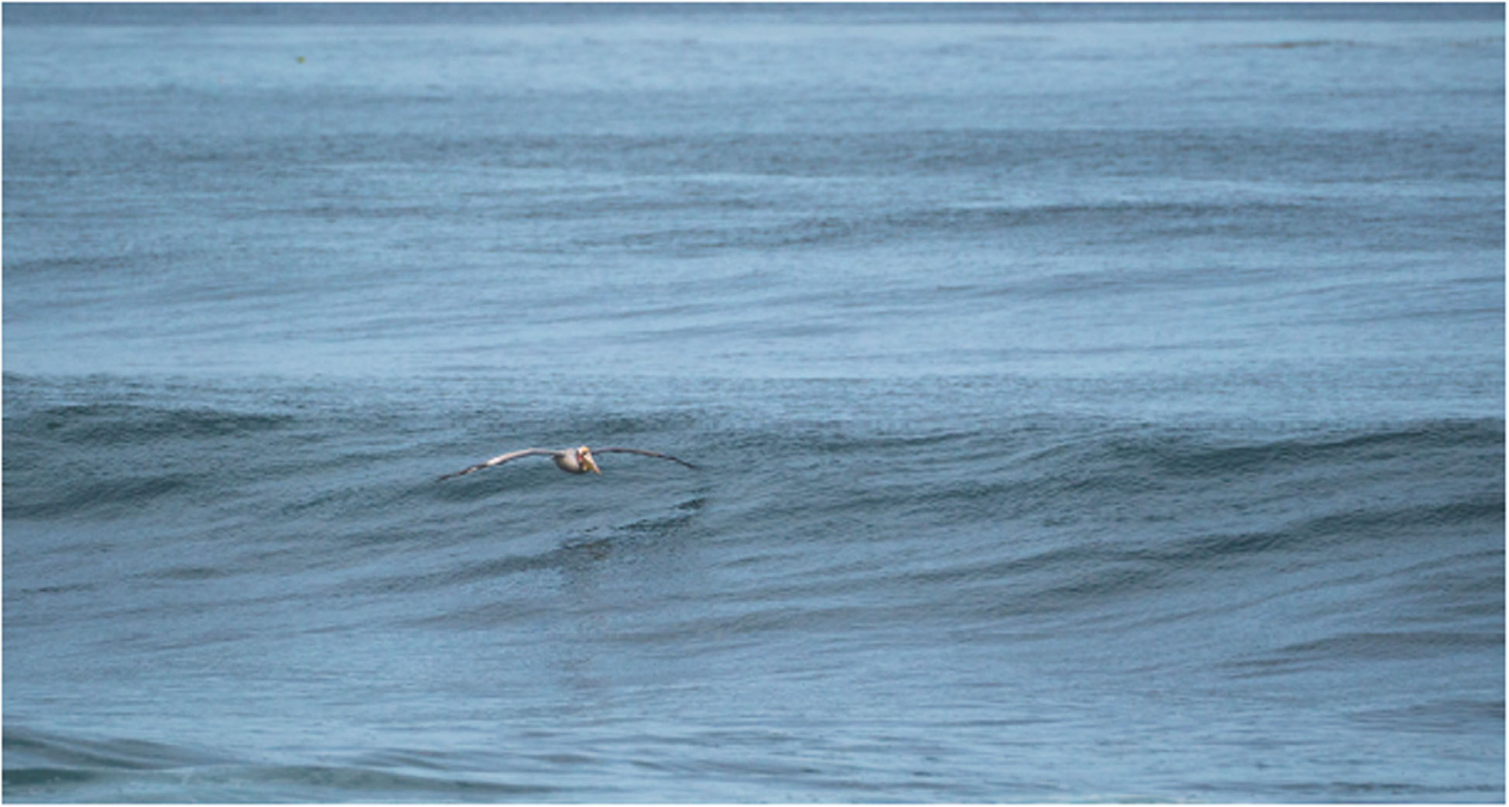
Photograph of a brown pelican using wave-slope soaring flight on a calm day in La Jolla, CA (Photograph: Simone Staff)

However, even in conditions with little to no ambient wind, albatrosses have been reported to track and follow waves on the ocean surface for long distances [7, 12]. At the coastline during calm conditions, pelicans can also be seen tracking the crests of shoaling waves just outside of the surf-zone, often in formation (see Fig. 1 and this example). In this fashion, they appear to be able to gain forward speed and thus kinetic energy, which they then convert to height, peeling off and upwards just as the wave begins to break. This altitude is then used to glide downwards and offshore to the subsequent approaching wave. By linking individual waves together, the birds can travel hundreds of meters or more with limited flapping.

Here we theoretically examine the possibility that the vertical component of the wind induced by traveling ocean waves [10] may explain the birds’ tendency to follow wave crests [3, 7, 10]. This behavior, which we term ‘wave-slope soaring,’ is shown by this analysis to have significant cost-benefit to energy efficient travel by comparison to steady level flight in and out of ground effect. It is a special case of ‘slope soaring’ flight with the primary difference that in wave-slope soaring, the updrafts are driven by traveling ocean surface waves [3, 7, 10, 13] pushing against a still atmosphere, rather than wind encountering a fixed object [2, 4].

## Methods

The goal is to estimate the energy savings associated with wave-slope soaring (WSS) flight. To accomplish this, we perform a theoretical study of the brown pelican practicing WSS over near-shoaling coastal waves. First, controls are developed in section Steady, level pelican flight in the absence of ocean waves. There we analyze the cost of steady, constant altitude pelican flight in the absence of ocean surface waves, out of ground effect (OGE, section Flight out of ground effect) and in ground effect (GE, section Flight in ground effect). In both cases, our description of the energetics uses energy consumption per distance travelled, or ‘cost of transport’ (COT) as the minimizing function [5]. These two results provide a baseline with which to compare the energy savings associated with wave-slope soaring flight, since flight in ground effect has demonstrated flight efficiency benefits [14–18].

Second, a description of the updrafts caused by near-shoaling waves is needed. In the air-sea interactions literature, any displacement of the atmosphere caused by traveling waves is known as ‘wave-induced wind’. The description of wave-induced wind is, in general, very complex due to the broad spectrum of ocean surface waves and nonlinear wave-wave and wave-wind interactions [19–22]. However, since wave-slope soaring behavior is seen in calm wind conditions close to the coastline in the brown pelican, and appears to favor smooth, long-crested swells, we proceed with a simplified model. This model describes wave-induced wind in zero ambient wind conditions offshore of the depth of wave-breaking (sections Airflow induced by near-shoaling waves, Potential flow over solitary waves). To retain some of the nonlinearities intrinsic to shoaling waves, but allow the problem to be analytically tractable, we assume a waveform shape of the well-studied soliton [23]. This approach has been effective in modeling near-shoaling, shallow water, ocean surface gravity waves, and was shown to be a reasonable representation of and ocean surface gravity wave in the region just outside of the surf-zone, where nonlinear steepening begins [23].

We use potential flow theory to model the wave-induced wind over these solitary waves. This is a significant simplification since, being an inviscid model, it does not account for development of boundary layers, especially on the trailing face of the moving wave. Obervations have shown that there are weak wind conditions where the atmospheric boundary layer remains laminar and well-attached [24–26], justifying the use of an inviscid assumption here. This assumption is violated in moderate and strong wind conditions, when a separated, turbulent boundary layer forms between wave-crests [27–30].

Since we observe the wave-slope soaring behavior in weak wind conditions, we assert for our model that the atmosphere is initially at rest, the ocean surface is smooth, the wave steepness is small, and the dynamics of the wave-induced wind in this idealized case can be largely captured by inviscid theory. Crucially, in what follows, we provide a comparison of the wave-induced wind produced by our potential flow model to measurements by [31] in section Potential flow over solitary waves.

Armed with the vertical component of the wave-induced wind from our inviscid model, we evaluate the cost of transport for flight through a moving medium following [5]. We then assess the efficiency of flight in WSS for a range of environmental/flight conditions, and compare to flight OGE and flight in GE to assess possible energy benefit of WSS flight (section Wave-Slope soaring flight). The physical characteristics of the brown pelican relevant to flight are drawn from Pennycuick [4] and given in Table 1.

**Table 1:** Average Brown Pelican Parameters [4]

Parameter	Symbol	Value
Mass	*M*	2.65 kg
Wingspan	*b*	2.10 m
Maximum Wing Area	$S_{w_{max}}$	0.45 m^2^
Wing Loading	$W/S_{w_{max}}$	57.8 N/m^2^
Aspect Ratio	*A*	9.8

### Steady, level pelican flight in the absence of ocean waves

We decompose the total aerodynamic drag into profile, parasitic, and induced drag components [32, 33]. Profile drag arises primarily from friction drag, and secondarily from pressure drag, both acting on the wings. Parasitic drag results primarily from pressure drag, and secondarily from friction drag, acting on the body. Finally, induced drag is a consequence of lift generation, associated with the downwash required to produce lift [5, 17, 32, 34]. From [32, 33], we write the total drag experienced by a bird gliding in still air at equilibrium as a function of airspeed *u*, such that

1 $$  D_{oge}(u) \approx \frac{\rho u^{2}}{2}\left(b\bar{c}C_{D_{pro}} + S_{b} C_{D_{par}} \right) + \frac{2k}{\pi\rho}\left(\frac{m g}{bu}\right)^{2},  $$

where *ρ* is air density (1.225 kg/m^3^), *b* is wingspan, $\bar {c}$ is the mean chord length, *S*_*b*_ is the body frontal area, *m* is the mass of the bird, *k* is the induced drag factor, and *g* is gravitational acceleration. $C_{D_{pro}}$ and $C_{D_{par}}$ are the profile and parasitic drag coefficients, respectively.

With units of [J/m], (1) can be interpreted as the COT for gliding flight in a still medium. In the case of gliding flight through a moving medium, (i.e. wind) [5] show that the theoretical COT ($\mathcal {C}$) can be expressed as

2 $$  \mathcal{C} \approx \frac{Du - mgw_{u}}{\sqrt{\left(u-w_{h}\right)^{2} + w_{s}^{2}}} = \frac{Du - mgw_{u}}{U},  $$

where *w*_*u*_ is the vertical component of the wind, *w*_*h*_ is the headwind experienced by the bird, and *w*_*s*_ is the crosswind experienced by the bird. Taylor *et al* [5] show that the denominator in (2) can be equivalently expressed as the groundspeed (*U*). Note that in the presence of ambient wind, *u*≠*U*. In the case where *w*→0, the airspeed and groundspeed equate (i.e. *u*→*U*) and (2) simplifies to (1). In section Wave-Slope soaring flight, we assess wave-slope soaring where the still-air initial condition is relaxed, necessitating the introduction of (2).

Beginning with the first term on the right hand side of (1), which represents the profile drag, we calculate the standard mean chord $\bar {c}$ assuming a straight tapered wing with constant area ($S_{w_{max}}$) and constant wingspan (*b*) given by Pennycuick [4] in Table 1. This calculation yields $\bar {c} = S_{w_{max}}/b = 0.21\hspace {0.1cm}\mathrm {m}$. Taylor & Thomas [34] propose setting $C_{D_{pro}} = 2.656\cdot \text {Re}^{-1/2}$, where $\text {Re} = \rho \bar {c} u/\mu $ is the chord Reynolds number, and *μ* is the dynamic viscosity of air (1.81·10^−5^ kg/m ·s). We will follow this assumption in our analysis to account for variation of the profile drag with changes in airspeed.

The second term on the right hand side of (1) represents the contribution from parasitic drag, which is difficult to estimate for seabirds [5]. Taylor & Thomas [34] suggest setting $\phantom {\dot {i}\!}S_{b} C_{D_{par}} = 0.01 \cdot S_{w_{max}}$. This formulation shows agreement with field estimates of the parasitic drag on diving passerines [35].

To characterize the induced drag, the third term in (1), we must assign a value to the induced drag factor, *k*. The induced drag factor is directly related to the wing shape [5,33,34,36]. The slotted tips of the brown pelican wing [4] act as winglets in tandem to reduce the induced drag experienced in gliding flight [36]. However, these winglets do not reduce *k* [37,38]. In Pennycuick’s model [33], a *k* value of 1.1 is used as default–Taylor & Thomas [34] show that only for an efficient elliptically loaded wing, rounding this factor down to *k*≈1 is appropriate. Accordingly, for a conservative estimate we will assume *k*=1.1.

#### Flight out of ground effect

To analyze cost-benefits of flight in ground effect (GE), we must first obtain a baseline comparison through analysis of the steady, constant altitude flight of a pelican in still air, out of ground effect (OGE). First, we assess the COT for a range of airspeeds (*u*) by using the parameters in Table 1 and section Steady, level pelican flight in the absence of ocean waves with (1) and evaluate for a range of values of airspeed *u* between [8,20] m/s for comparison with [4,39]. We refer to the airspeed that minimizes the COT as the minimum-cost velocity, denoted *u*_*mc*_. Second, we quantify the required power output by the bird for a given COT. This is done by multiplying the COT with the corresponding airspeed, as the gliding flight here is apprioximated to be level.

Thus, power expenditure out of ground effect as a function of airspeed, *P*_*oge*_(*u*), can be written as

3 $$  {}P_{oge}(u) \approx \frac{\rho u^{3}}{2}\left(b\bar{c}C_{D_{pro}} + S_{b} C_{D_{par}} \right) + \frac{2k}{\pi\rho u}\left(\frac{m g}{b}\right)^{2}.  $$

**Fig. 2:**
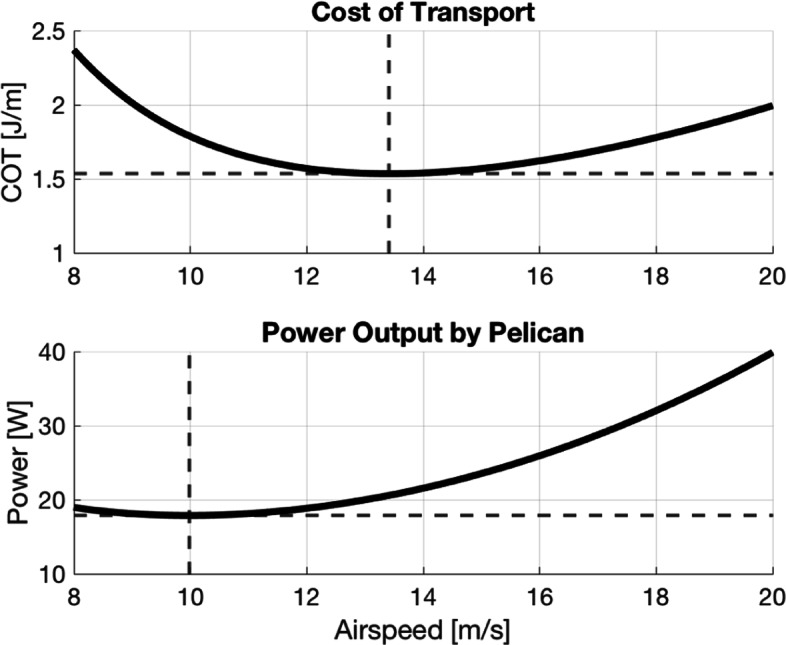
Efficiency of steady, level flight out of ground effect in absence of ocean waves. Top Panel: COT as a function of airspeed for steady, level flight out of ground effect (OGE). Minimum-cost velocity ∼13.4 m/s with a corresponding COT of ∼1.5 J/m. Bottom Panel: Power output as a function of airspeed for steady, level flight OGE. Minimum-power velocity ∼10.0 m/s with a corresponding power output of ∼17.9 W

The airspeed that minimizes the required power output will be referred to as the minimum-power velocity, denoted *u*_*mp*_. The results of this analysis are displayed in Fig. 2. COT and power output as functions of airspeed are shown in the top and bottom panels, respectively.

Using the values corresponding to the brown pelican as given in section Steady, level pelican flight in the absence of ocean waves and the density and viscosity of air at sea level, we find that *u*_*mc*_∼13.4 m/s with a corresponding COT calculated from (1) of ∼1.5 J/m. Though *u*_*mc*_ provides the minimum COT, this value exceeds *u*_*mp*_∼10.0 m/s, which is calculated by minimizing (3) with respect to velocity.

The precise measurement of the metabolic power output for brown pelican flight is not readily available in the literature. Noting that the mean airspeed from [4] lies roughly halfway between *u*_*mc*_ and *u*_*mp*_, we estimate the range of power expenditure for the brown pelican to be roughly [*P*(*u*_*mp*_), *P*(*u*_*mc*_)] for steady, level flight. Using (3), we calculate this range of expected power output to be ∼[17.9, 20.6]. W. Ballance [40], in study of the red footed booby, a smaller marine bird, found that it expends an average of ∼20 W in gliding flight. Though further experiment will be required to verify our estimated power requirement of the brown pelican in steady, level flight, we use this minimum COT as the primary control for what follows.

#### Flight in ground effect

Flight in ground effect decreases the induced drag, which is commonly referred to as ‘induced drag savings’ [14]. This is estimated using a drag reduction factor, *ϕ*, that is a function of flight height *H* and wingspan *b*. The profile and parasitic drag, which are related to the form of the flier only, remain unchanged [14*–*17]. Ground effect occurs when *ϕ*<1 for heights less than one wingspan (*H*<*b*) and tends to *ϕ*≈1 as *H*>*b* [15,16].

An analytical expression for *ϕ* is given by [16], which is written as

4 $$  \phi = \frac{1 - 2/\pi + (16H/\pi b)^{2}}{1 + (16H/\pi b)^{2}}.  $$

Including this factor in (1) gives an expression for the total drag experienced in GE, written

5 $$  {}D_{ge}(u) \approx \frac{\rho u^{2}}{2}\left(b\bar{c}C_{D_{pro}} + S_{b} C_{D_{par}} \right) + \phi\frac{2k}{\pi\rho}\left(\frac{m g}{bu}\right)^{2}.  $$

**Fig. 3:**
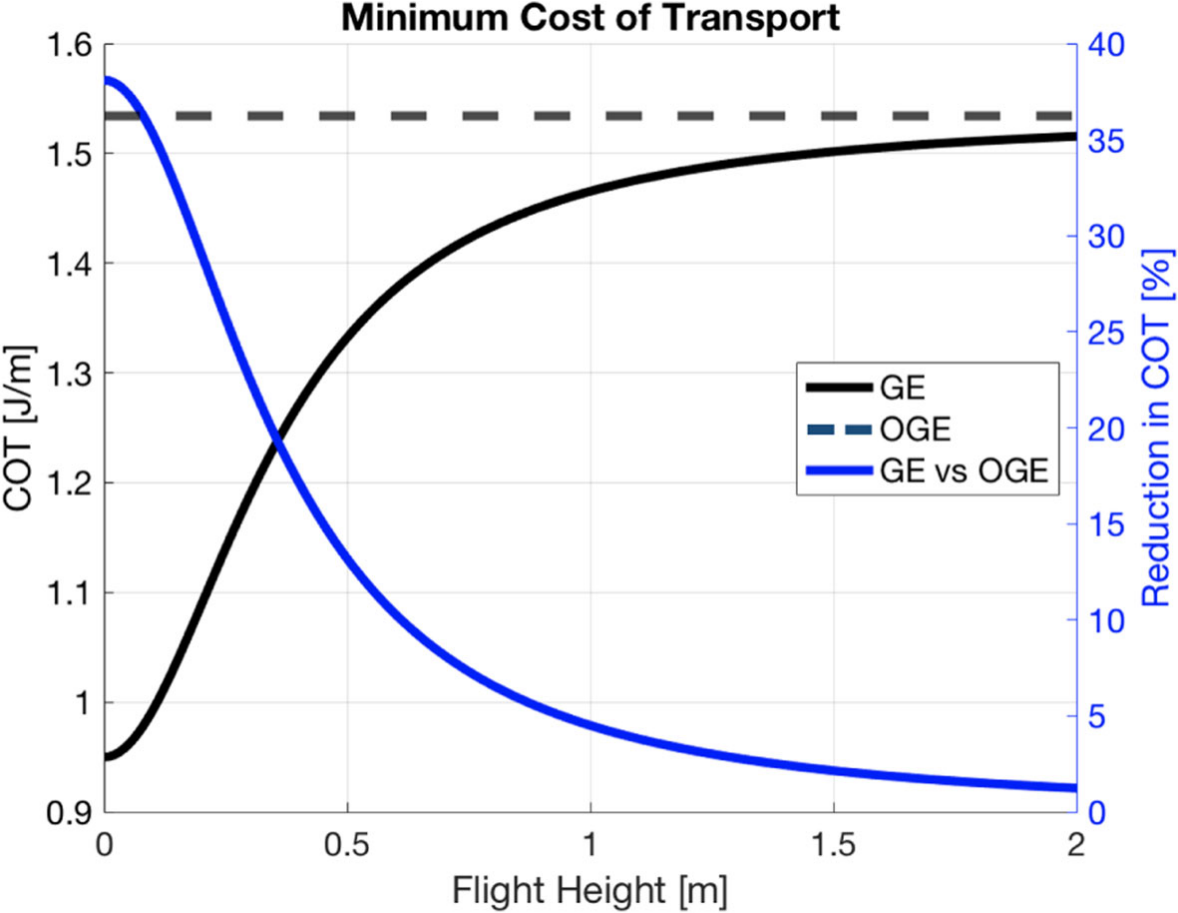
Using (5) we calculate the energetics of flight in GE. Minimum cost of transport as a function of flight height is plotted on the left hand y-axis, in black. Reduction in minimum cost of transport as a function of flight height is plotted on the right hand y-axis, in blue. Note that this compares flight at different airspeeds, as GE reduces the minimum cost velocity [17]

Noting again that for flight in still air the COT equates to the drag, we use (5) to calculate COT in GE for flight heights between 0 to 2m above the sea surface, which corresponds to *H*/*b*≈1 where the GE becomes negligible. These results are shown in Fig. 3.

Hainsworth [14] reports average brown pelican ground-effect flight height of 33 cm, with a standard deviation of 5 cm. For flight heights on the range reported by [14], COT required for flight is ∼1.1-1.3 J/m, with corresponding percent mechanical advantages of ∼15-25%, when compared to the 1.5 J/m required for flight OGE. These values agree with [17,18].

### Airflow induced by near-shoaling waves

As ocean waves translate, they induce airflow as a result of the no-penetration condition on a boundary, even in the case where there is no ambient wind. Interestingly, wave-induced wind might have been first reported in 1925 by Idrac [10] in his study of albatrosses, who noted that large, steep traveling ocean surface gravity waves can produce updrafts with vertical velocity in excess of 2 m/s at 8 meters height. It was also noted that these updrafts effects can be felt up to 15 meters above the ocean surface [10].

Research on wave-induced wind has principally focused on its impacts on the ocean and atmospheric boundary layer properties and dynamics. These include both numerical (e.g. [41]) and observational studies (e.g. [28–30]). Recently, the upward transfer of momentum from ocean swell to the wind was experimentally verified by [31] in their experiment aboard the Scripps Institution of Oceanography’s FLoating Instrumentation Platform (R/P FLIP). Using wave measurement apparatus and an array of ultrasonic anemometers, they estimated wave-induced components of the wind velocity for various wind-wave conditions, producing an empirical curve for wave-induced wind components scaled by surface wave orbital velocity as a function of height above the ocean scaled by surface wave wavenumber [31].

We aim to model the process of wave-slope soaring in the coastal ocean offshore of the surf-zone during periods of weak winds, when it is most commonly observed. In this region, the ocean surface waves are depth limited and thus modified from a linear sinusoidal state. It is typical to model ocean gravity waves just offshore of the surf-zone as solitons [23]. Solitons are localized nonlinear waves that propagate without change of speed or form [42]. Here we assume weak nonlinearity such that we can use the Korteweg-de Vries (KdV) equation [23]. Note that the KdV equation is valid only for waves in shallow water with *λ*≫*h* or equivalently *kh*≪1, where *λ* is the wavelength, *k* is the wavenumber, and *h* is the ocean depth.

Neglecting surface tension, we begin with the dimensional traveling soliton solution,

6 $$  \eta(x,t) = A\hspace{0.1cm}\text{sech}^{2}(kx-\omega t),  $$

where

7 $$  \begin{aligned} A &\equiv \frac{4}{3}\hspace{0.1cm}k^{2}h^{3}, \\ \omega &\equiv k\sqrt{gh}-\frac{2}{3}\hspace{0.1cm}k^{3}h^{2}\sqrt{gh}. \end{aligned}  $$

We use the dispersion relation to write the wave’s dimensional phase velocity (*c*) as

8 $$  c = \sqrt{gh}\left[1-\frac{2}{3}(kh)^{2}\right].  $$

Our definition for the amplitude in (7) can be used to eliminate *k* from (8), allowing us to write the phase velocity as a function of amplitude and depth,

9 $$  c = \sqrt{gh}\left[1 - \frac{A}{2h}\right].  $$

We now use the dispersion relation and the definition of amplitude given in (7) to write an expression for the period as a function of amplitude and depth,

10 $$  T = \frac{4\pi h}{\sqrt{3gA}\left(1 - \frac{A}{2h}\right)}.  $$

For the waves we consider in the case of WSS flight (section Wave-Slope soaring flight) with height of 2 meters and period of 15 seconds, the soliton approximation is valid over depths on the order of 10 m, corresponding in our local area to distances of 100 to several hundred meters offshore of the surf zone.

### Potential flow over solitary waves

The use of potential flow solutions requires that the fluid be irrotational, incompressible, and inviscid within our region of interest. The symmetry of the solitary waveform we have imposed justifies the assumption of irrotational flow, while the assumption of incompressible flow is justified by the small Mach number at ocean surface wave velocities. However, as noted in section Methods, the atmospheric boundary layer over the ocean is generally turbulent in moderate to strong winds. This restricts our analysis to weak or no wind conditions. Furthermore, the low amplitude, smoothly varying waveform used here is meant to approximate near-shoaling swell in no wind conditions, where flow separation of the wave-induced wind field is unlikely [24*–*27].

We model potential flow over the soliton

11 $$  \eta = A\hspace{0.1cm} \text{sech}^{2}(kx),  $$

moving at phase speed *c*. We first boost to a frame of reference moving with the soliton such that *U*_*∞*_=−*c*. As we are assuming potential flow conditions, the system is governed by Laplace’s equation for the stream function,

12 $$  \Delta \psi = 0,  $$

with the no-penetration boundary condition

13 $$  \left[\mathbf{u}\cdot\hat{\mathbf{n}} = 0 \right]_{z = A\hspace{0.1cm} \text{sech}^{2}(kx)}.  $$

Laplace’s equation in the upper half plane with a no-penetration boundary condition on the horizontal axis is a well-studied problem that can be solved using Green’s theorem. With the proper nondimensionalization, the soliton boundary can act as a small disturbance, or ‘perturbation’ to this problem. Thus, we will use perturbation theory to derive an approximate solution for the airflow over a soliton.

We introduce the nondimensional coordinates

14 $$  \zeta = kz, \hspace{2cm} \xi = kx.  $$

From (7) we can write

15 $$  Ak = \frac{4}{3} (kh)^{3}.  $$

As the soliton was derived in the limit of *λ*≫*h*, it follows that *kh*≪1. We will define another nondimensional coordinate *ε*≡*Ak* such that *ε*∝(*kh*)^3^≪1. This coordinate, *ε*, will serve as the small disturbance upon which we build our perturbation expansions. Using (11), (14), and *ε* we can express the boundary in terms of nondimensional coordinates as

16 $$  \zeta = \epsilon \hspace{0.1cm} \text{sech}^{2}(\xi).  $$

Since *ε*≪1 in the scaled geometry, to a first approximation we simply have to solve Laplace’s equation in the upper half plane.

By the definition of the stream function, we have

17 $$  u_{wi} = \frac{\partial \psi}{\partial \zeta}, \hspace{1cm} w_{wi} = -\frac{\partial \psi}{\partial \xi}.  $$

In this case, the stream function *ψ* is a function of the spatial variables *ξ* and *ζ*, as well as the scaled wave dimension *ε* such that *ψ*=*ψ*(*ξ*,*ζ*,*ε*). The boundary condition (13) enforces that *ψ* must be constant everywhere on the sea surface. Integrating (17 a), using the condition that as *ξ*→± *∞*,*ψ*→−*c* *ζ*, and taking *ψ* to be constant on the sea surface gives the condition

18 $$  \psi(\xi,\epsilon\hspace{0.1cm}\text{sech}^{2}\xi) = 0.  $$

With *ε*≪1, we Taylor expand (18). This gives

19 $$  \psi(\xi,0) + \epsilon\hspace{0.1cm}\text{sech}^{2}(\xi)\psi_{\zeta}(\xi,0) + O\left(\epsilon^{2}\right) = 0  $$

where subscripts denote partial derivatives. We now expand *ψ* in a regular perturbation expansion to the order of *ε*, yielding

20 $$  \psi = \psi_{0}+\epsilon\hspace{0.1cm}\psi_{1} + O\left(\epsilon^{2}\right),  $$

where for all *ψ*_*n*_, with *n*∈[0,*∞*),*ψ*_*n*_=*ψ*_*n*_(*ξ*,*ζ*) and *Δψ*_*n*_=0. At *O*(*ε*^0^),*Δψ*_0_=0. Integration yields

21 $$  \psi_{0} = -c\hspace{0.1cm}\zeta.  $$

Substitution of our perturbation expansion (20) with (21) into (19) gives

22 $$  \epsilon\left[\hspace{0.1cm}\psi_{1}(\xi,0) -c\hspace{0.1cm}\text{sech}^{2}(\xi)\hspace{0.1cm}\right] + O\left(\epsilon^{2}\right) = 0.  $$

At *O*(*ε*) in (22) we obtain the boundary condition

23 $$  \psi_{1}(\xi,0) = c \hspace{0.1cm}\text{sech}^{2}(\xi),  $$

necessary to solve the Laplacian at *O*(*ε*),*Δψ*_1_=0. By Green’s theorem, the solution to an arbitrary partial differential equation can be expressed as an integral of the relevant Green’s function, provided such a function exists [43]. This allows us to solve for the *O*(*ε*) term of the stream function (*ψ*_1_) using the Green’s function for Laplace’s equation in the two-dimensional upper half plane with the Dirichlet boundary condition in (23). This particular Green’s function can be written as

24 $$  \begin{aligned} G\left(\xi,\zeta;\xi^{\prime},\zeta^{\prime}\right) = &\frac{1}{2\pi}\Bigg(\text{ln}\sqrt{\left(\xi-\xi^{\prime}\right)^{2} + \left(\zeta-\zeta^{\prime}\right)^{2}} \\ & - \text{ln}\sqrt{\left(\xi-\xi^{\prime}\right)^{2} + \left(\zeta+\zeta^{\prime}\right)^{2}}\Bigg), \end{aligned}  $$

[43] where (*ξ*^′^,*ζ*^′^) lies within the upper *ξ*- *ζ* plane.

Using (23) and (24) with Green’s theorem allows us to obtain an expression for *ψ*_1_(*ξ*,*ζ*) as

25 $$  \psi_{1}(\xi,\zeta) = \frac{c}{\pi}\int_{-\infty}^{\infty} \frac{\zeta\hspace{0.1cm}\text{sech}^{2}(\xi^{\prime})}{(\xi - \xi^{\prime})^{2} + \zeta^{2}}\hspace{0.1cm}d\xi^{\prime},  $$

where *ξ*^′^ is the variable of integration. We remove the singularity by dividing the domain of integration at *ξ*^′^=*ξ*. Combining (25) and (21) with (20), we can now obtain a full expression for *ψ* as

26 $$  \begin{aligned} \psi = -c\zeta &+ \frac{Akc}{\pi}\int_{0}^{\infty} \frac{\zeta}{\xi^{\prime2}+\zeta^{2}}\Big[\text{sech}^{2}(\xi - \xi^{\prime}) \\ &+\hspace{0.1cm} \text{sech}^{2}(\xi + \xi^{\prime})\Big]d\xi^{\prime} + O(\epsilon^{2}), \end{aligned}  $$

where *ξ*^′^ remains our variable of integration. This expression can now be evaluated numerically.

Using (17 a), we can carry out the differentiation to obtain an integral for the horizontal wave-induced flow speed *u*_*wi*_ in the frame of reference moving with the wave to the order of *ε* in terms of scaled coordinates as

27 $$  {}\begin{aligned} u_{wi} = -c &+ \frac{Akc}{\pi}\hspace{0.1cm}\int_{0}^{\infty}\frac{\xi^{\prime2} - \zeta^{2}}{(\xi^{\prime 2} + \zeta^{2})^{2}}\Big[\text{sech}^{2}\left(\xi - \xi^{\prime}\right) \\ &+\hspace{0.1cm} \text{sech}^{2}(\xi + \xi^{\prime})\Big]\hspace{0.1cm}d\xi^{\prime} + O\left(\epsilon^{2}\right), \end{aligned}  $$

where we have substituted the definition of *ε* (*ε*≡*Ak*) back into the expression. Similarly, we can use (17 b) to write an integral for the vertical wave-induced flow speed *w*_*wi*_ to *O*(*ε*) in terms of scaled coordinates as

28 $$  {}\begin{aligned} w_{wi} = \hspace{0.1cm}&\frac{2Akc}{\pi}\hspace{0.1cm}\int_{0}^{\infty}\frac{\zeta}{\xi^{\prime2} + \zeta^{2}}\left[\text{sech}^{2}(\xi - \xi^{\prime})\text{tanh}\left(\xi - \xi^{\prime}\right)\right. \\ &\left.+\hspace{0.1cm} \text{sech}^{2}(\xi + \xi^{\prime})\text{tanh}(\xi + \xi^{\prime}) \right]\hspace{0.1cm}d\xi^{\prime} + O\left(\epsilon^{2}\right). \end{aligned}  $$

**Fig. 4:**
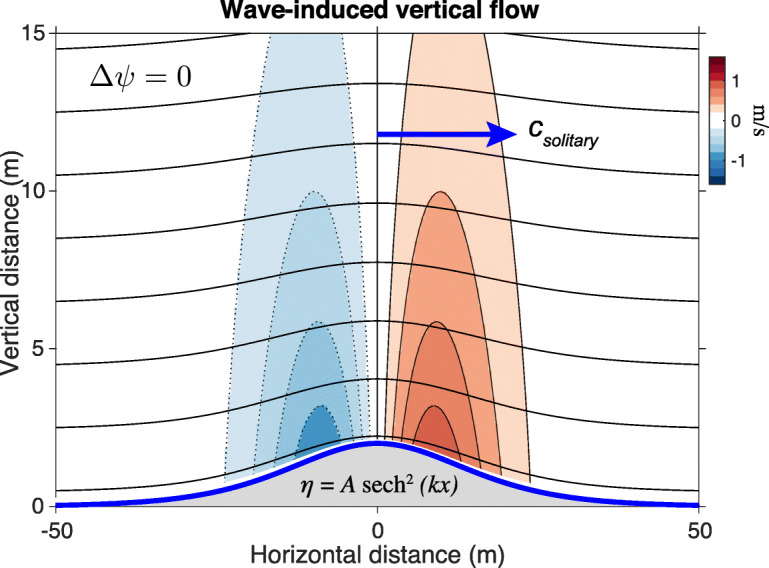
Flow visualization of the wave-induced updraft over the soliton *η*(*x*)=*A* sech^2^(*kx*) in the *x*−*z* plane, forced by the Laplacian, *Δψ*=0. The soliton shown has wave height *A*=2 m, period *T*=15 s, and travels at phase speed *c*_*solitary*_≈7.75 m/s

In order to evaluate the flow velocities, we need the wavenumber *k*. As the nonlinearities intrisic to KdV solitons are captured in our expressions for phase velocity (9) and corresponding wave period (10), the linear dispersion relation can be used to obtain an expression for the wavenumber in terms of the phase velocity and period as *k*=2*π*/*cT* [23]. Note that (9) and (10) can be co-evaluated to produce the phase velocity of our model wave for a given wave height (*A*) and period (*T*). Following this analysis, the resultant phase velocity, wavenumber, and given amplitude can be inserted into (27) and (28), yielding the theoretical wave-induced wind in the near-shoaling regime relevant to wave-slope soaring. A visualization of this flow field above a propagating, weakly nonlinear surface wave with no ambient wind is displayed in Fig. 4.

**Fig. 5:**
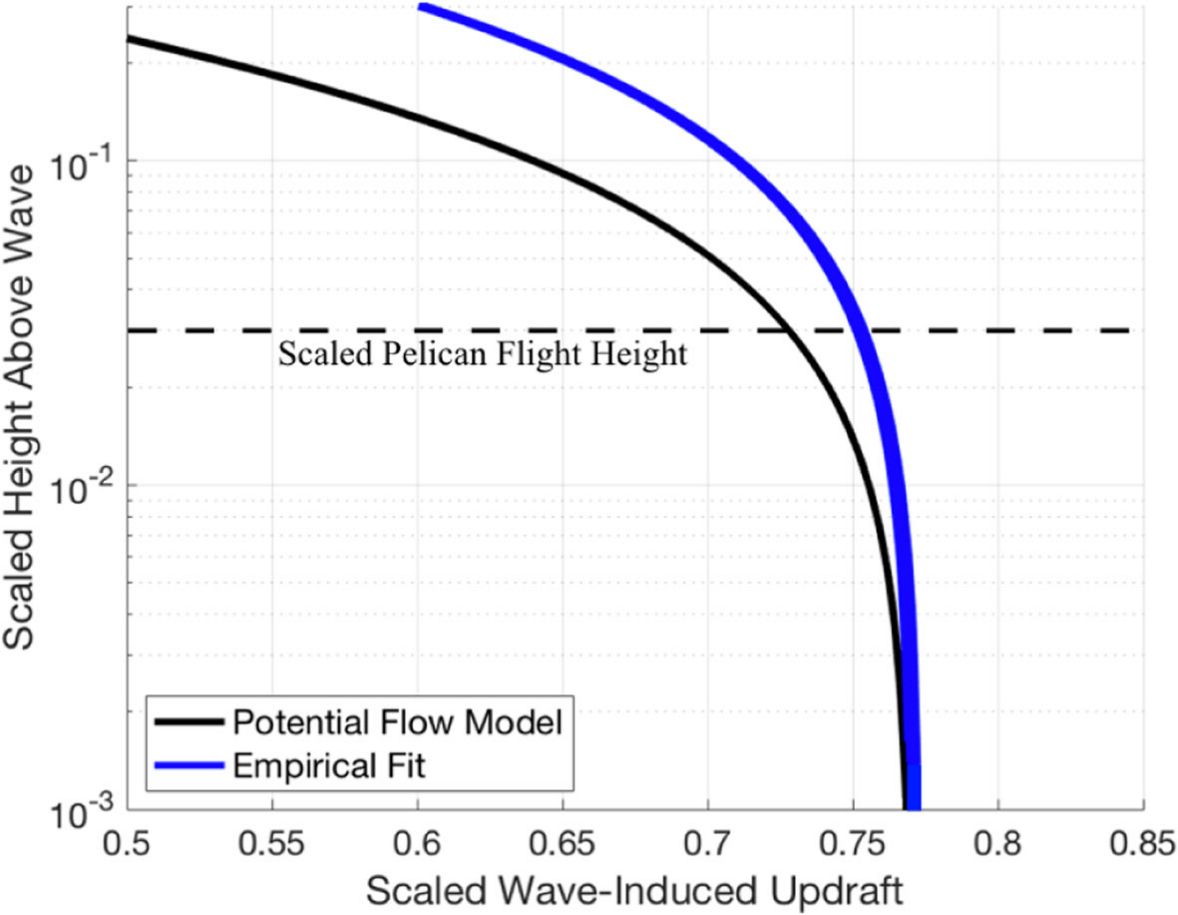
Comparison of potential flow theory to measurements by Grare et al. [31]. The vertical component of the wave-induced wind (*w*_*wi*_) scaled with surface orbital velocity (*Akc*) is shown on the x-axis and scaled height (*kz*) is shown on the y-axis in a semilog plot. We see that the potential flow estimate for the vertical component of the wave-induced wind gives a slight under-prediction but generally agrees with the empirical model best-fit curve for the vertical component of wave-induced wind from [31]. This comparison to observations gives confidence that the wave-induced wind estimates used here are reasonably representative of the conditions at sea during wave-slope soaring

To validate our expression for wave-induced wind, we compare our predictions to the findings of Grare et al. [31] (Fig. 5), using the vertical component of the wave-induced wind nondimensionalized by surface orbital velocity (*Akc*), and the previously defined nondimensional vertical height *ζ*=*kz*. In our region of interest (*kz*≤0.05), we see good agreement with an empirical fit to the measurements given by Grare et al. as

29 $$  w/Akc = 0.85\Big[1 - 0.66\cdot \text{exp}\Big(-\Big|\frac{c}{u}-1\Big|\Big)\Big]\text{exp}(-0.83\cdot kz),  $$

with coefficient of determination *r*^2^=0.76. (Fig. 5 [31]).

### Wave-Slope soaring flight

**Fig. 6:**
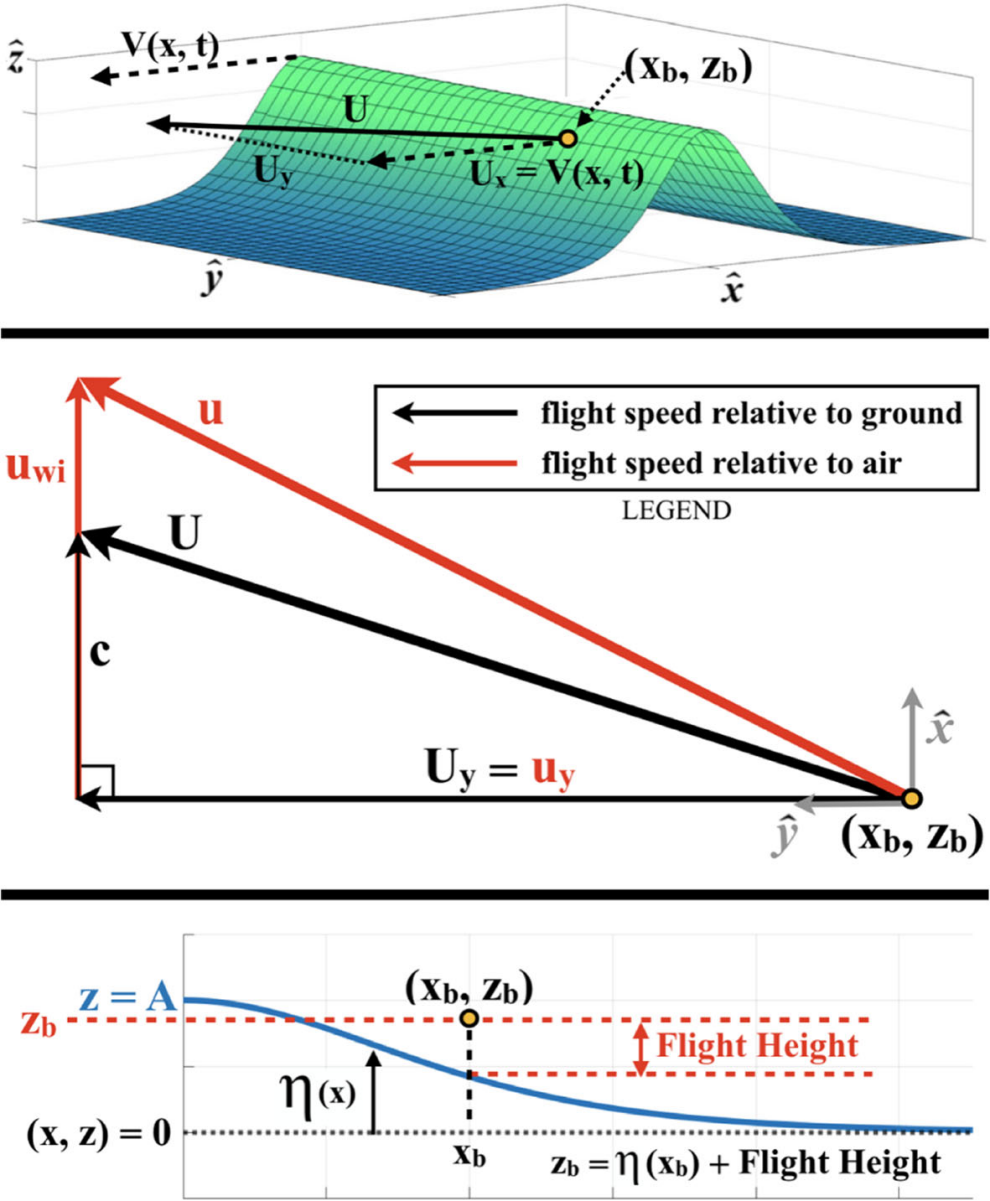
Top Panel: Coordinate system for the inertial trajectories of a pelican wave-slope soaring on a solitary wave. Middle Panel: Ground velocity vs. air velocity for wave-slope soaring and respective relations to phase velocity (*c*) and wave-induced wind (*u*_*wi*_). Bottom Panel: Coordinate system for (*x*_*b*_,*z*_*b*_) in terms of *η*(*x*) and bird’s flight height

We assume that the only wind-field is that which is driven by the wave. We define coordinates such that $\hat {\mathbf {x}}$ is in the direction of wave propagation, $\hat {\mathbf {y}}$ is parallel to the wave front, and $\hat {\mathbf {z}}$ is in the vertical direction. A schematic of this coordinate system is shown in the top panel of Fig. 6. In order to gain benefit from the wave for extended periods of time, the bird must translate in $\hat {\mathbf {x}}$ so that its groundspeed in the direction of wave propagation (*U*_*x*_) will match the phase velocity of the wave, *V*(*x*,*t*). The phase velocity of the wave is constant under KdV soliton theory so that *V*(*x*,*t*)=*c*, which we calculate for specified wave height and period using (9) and (10).

In setting *U*_*x*_=*c*, it follows from section Potential flow over solitary waves that the bird’s airspeed in the direction of wave propagation (*u*_*x*_) must then equate to the horizontal component of the wave-induced wind (*u*_*wi*_) at the bird’s location, shown in the middle panel of Fig. 6. Because the components of groundspeed and airspeed in the along-wave-crest direction are equal (i.e. *U*_*y*_=*u*_*y*_, Fig. 6, middle panel), we can use the system’s geometry to eliminate *u*_*y*_ and *U*_*y*_. This allows us to write an expression for the groundspeed in terms of airspeed (*u*), phase velocity (*c*), and the horizontal component of the wave-induced wind (*u*_*wi*_) as

30 $$  U = \sqrt{u^{2} + c^{2} - u_{wi}^{2}}.  $$

In (30), *u*_*wi*_ is a function of the spatial flight coordinates (*x*_*b*_,*z*_*b*_) i.e. *u*_*wi*_=*u*_*wi*_(*x*_*b*_,*z*_*b*_).

To estimate (*x*_*b*_,*z*_*b*_), we impose the assumption that the bird will fly at the optimal location in the space above the wave for minimizing COT, and will remain at this location throughout soaring flight. Section Potential flow over solitary waves shows that the optimal flight location is directly over the inflection point of the wave surface, where the slope is the steepest. Accordingly, for the *x* coordinate of the bird’s center of mass (*x*_*b*_), we find the point of maximum slope associated with the waveform developed in section Airflow induced by near-shoaling waves. For the *z* coordinate of the bird’s center of mass (*z*_*b*_), we use the free surface elevation at this point of maximum slope, *η*(*x*_*b*_), calculated from (6) and add the case-respective flight height. A schematic of this procedure is displayed in the bottom panel of Fig. 6

As the wavelength is large compared to the wingspan of the bird and the wave slope is small, we ignore variation of the wave-induced wind over the wingspan of the bird. The updraft component of the wind-field in (2) is driven solely by the wave, which with neglecting variation over wingspan justifies the use of a single value for *w*_*u*_, as *w*_*u*_=*w*_*wi*_, in (2). Using the formalism developed in section Potential flow over solitary waves, *w*_*wi*_ is calculated from (28) using the coordinates (*x*_*b*_,*z*_*b*_) and case-respective wave parameters. Together with the substitution of (30) into (2) and using (27) to evaluate *u*_*wi*_(*x*_*b*_,*z*_*b*_), we estimate the COT in wave-slope soaring, denoted $\mathcal {C}_{wss}$. This can be expressed as

31 $$  \mathcal{C}_{wss} \approx \frac{Du - mgw_{wi}}{\sqrt{u^{2}+c^{2} -u_{wi}^{2}}},  $$

where *D*, *u*_*wi*_, and *w*_*wi*_ are given by (1), (27), and (28) respectively, while *c* is given by evaluating (10) for *h* and substituting into (9).

**Fig. 7:**
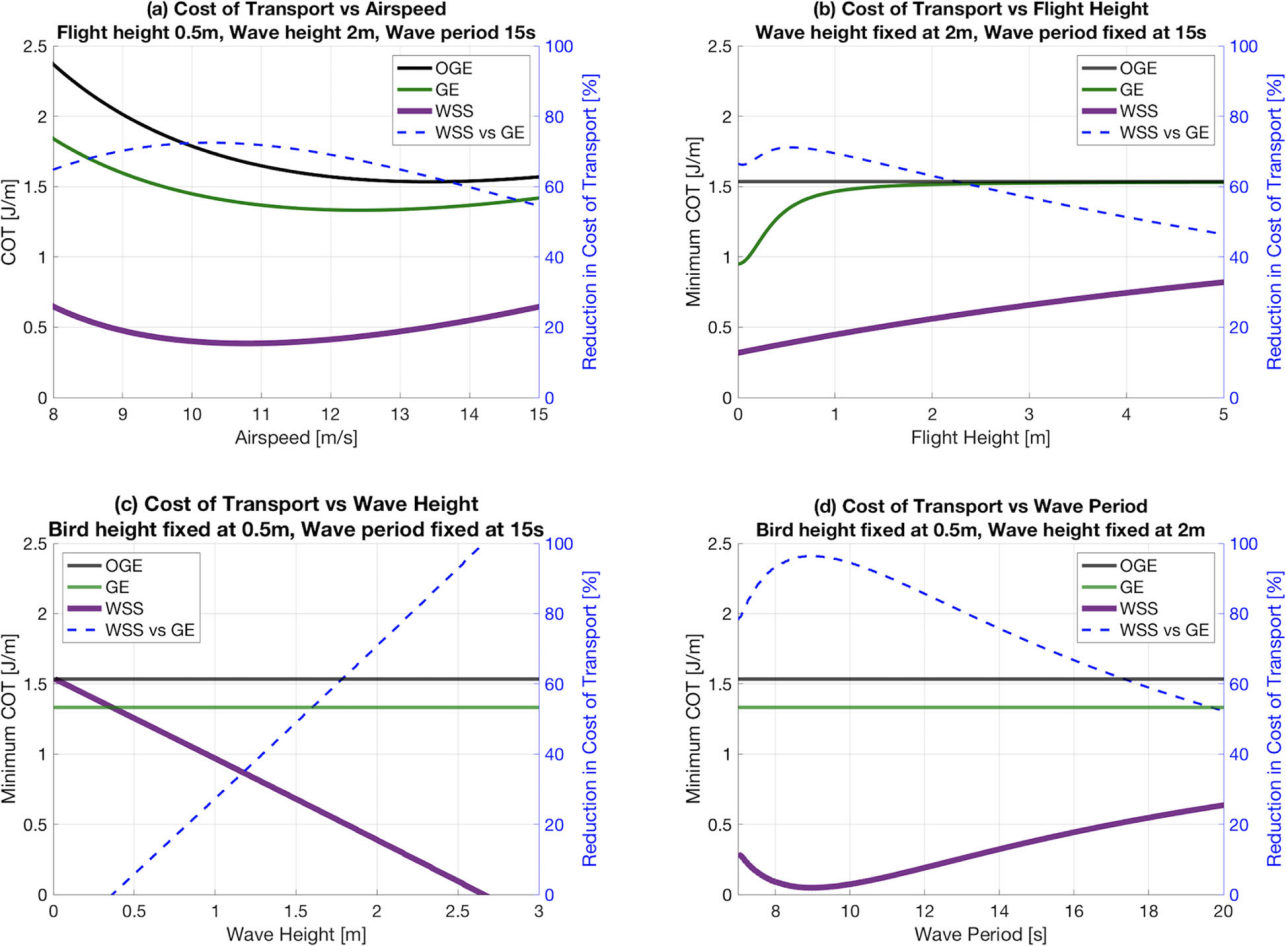
We explore the efficiency of wave slope soaring flight (WSS) under a range of environmental/flight conditions. In each panel, the corresponding percent reduction relative to flight in ground effect (GE) is shown in blue, with the right hand y-axis. **a** Cost of transport (COT) of WSS is shown for a range of airspeeds consistent with [4, 39] in the default case of 0.5m flight height, 2m wave height, 15s wave period. **b** Minimum COT of WSS over a wave of 2m height, 15s period, varying flight height. **c** Minimum COT of WSS for 0.5m flight height over 15s period waves, varying wave height. **d** Minimum COT of WSS for 0.5m flight height over 2m high waves, varying wave period

Thus, we see that, for the simplifying assumptions we have made here, and ignoring ground effect, we have an expression for the COT in WSS as a function of airspeed, wave height, wave period, flight location, and bird geometry, i.e.

32 $$  \mathcal{C}_{wss} = \mathcal{C}_{wss}(u,A,T,x_{b},z_{b},\text{bird geometry}).  $$

and is shown for a range of parameters in Fig. 7.

## Results

From our control, we find the minimum COT in steady, constant altitude pelican flight out of ground effect ∼1.5 J/m with a corresponding minimum-cost velocity of ∼13.4 m/s (Fig. 2). When we consider ground effect flight for heights in the range reported by [14], we find the COT is reduced by ∼15-25% to 1.1-1.3 J/m and the minimum-cost velocity is decreased to ∼12 m/s (Fig. 3). A test case of WSS over a wave of 2 meters height with a 15 second period is used for consistency with a typical Southern California winter swell event [44]. Under these conditions we estimate large increases in energy savings in comparison to the control cases. Ignoring any benefit from GE, we find reductions in COT on the order of 70% for flight at 0.5m height, as compared to the 15% cost-benefit from GE at this height, shown in panel (a) of Fig. 7. As expected, for lower flight heights and larger waves, the COT and minimum-cost velocities are even further reduced. This is shown in panels (b) and (c) of Fig. 7, respectively. Increasing the wave period does not monotonically decrease the COT. Though increasing the period increases the wave speed, it decreases the wave steepness, resulting in a nonlinear relationship between the wave’s phase velocity and the updraft, headwind, and crosswind experienced by the bird. The result is a maximum updraft at a relatively short period for a given wave height, shown in panel (d) of Fig. 7.

## Discussion

In the simplified case studied here, we show theoretically that wave-slope soaring can provide a considerable reduction in COT relative to steady, level flight in and out of ground effect. This may account for the widespread use of the behavior in the brown pelicans that live in the coastal waters of Southern California.

There are several limitations of the theory presented here. We used the simplifying assumption of a weakly nonlinear solitary waveform. In reality, when observing pelicans employing wave-slope soaring, it is common for them to soar well into the surf-zone, where nonlinearities become progressively stronger. In this regime, it becomes unreasonable to approximate the waveform as a soliton, and a more elaborate theory or numerical simulation would need to be employed. Added complications in the real world include the directional and frequency spread that characterizes ocean swell [45–48], and that shoaling waves tend to arrive in groups [49–52], meaning that isolating the effect of a single wave may ignore an important effect of a train of waves shoaling in sequence.

The assumption of no ambient airflow in our model is another significant simplification. Our solution is framed around the benefit of vertical flow in the atmosphere perturbed by a travelling wave, which would be altered as ambient wind speeds increase, potentially increasing vertical velocities near the wave face [53]. However, the development of a turbulent boundary layer that tends toward separation between wave-crests is known to occur in moderate and strong wind scenarios [27, 54, 55]. This renders the inviscid simplification that underpins the potential flow solution invalid.

Perhaps turbulent airflow over ocean waves is not amenable to wave-slope soaring. In the Southern Ocean, wave-slope soaring in albatrosses is only observed during rare calm periods [7]. This suggests that as the boundary layer becomes turbulent, dynamic soaring is a more effective strategy. A separated, turbulent boundary layer may be detrimental to wave-slope soaring since updrafts associated with traveling waves may be reduced in magnitude or lose coherence in time or along the wave crest, which is the primary direction of travel.

High-resolution numerical simulations capable of representing the full response of the atmosphere to ocean waves are now being used to study air-sea interactions [19, 22, 41, 56, 57]. These simulations, if verified by future field observations, allow a 3-D and time varying wind field to be calculated for different forcing scenarios that would be of great utility for examining the aerodynamics of wave-slope soaring. Similarly, individual brown pelicans tagged with inertial measurement units and fast-rate GPS positions would allow for the flight behavior of wave-slope soaring to be better quantified [58–60]. In particular, time-series measurements of accelerations and airspeed could be used to quantify the forces acting during wave-slope soaring behavior [58, 61–63]. Further investigation of wave-slope soaring is not relevant only to the ecology of seabirds, but in the future may be one of a suite of environmental scenarios in which unmanned aerial vehicle control systems can maximize flight endurance using environmental energy [64–68].

## Conclusions

The theoretical framework presented here suggests that brown pelicans could reduce the energetic demands of gliding flight by ∼60-70% via utilizing wave-slope soaring during periods of weak winds. Although there must be some risk associated with flying at a relatively high speed very close to an undulating and evolving surface, the benefit in terms of efficiency apparently favors the behavior. Surfing of shoaling and breaking waves has been documented in several species of marine mammals, wherein it is assumed that the activity represents play [69]. Brown pelicans, on the other hand, may leverage their ability to ride waves for long-distance travel, since flight allows them to connect a set of multiple shoaling waves in sequence. This allows continuous wave-riding for periods of minutes, and may account for travel of kilometers up or down the coast. Cost-effective travel resulting from wave-slope soaring behavior may have an important impact on the foraging range and foraging strategy of these ecologically important creatures.

## Data Availability

Not applicable.
